# A Low-Cost Metamaterial Sensor Based on DS-CSRR for Material Characterization Applications

**DOI:** 10.3390/s22052000

**Published:** 2022-03-04

**Authors:** Waseem Shahzad, Weidong Hu, Qasim Ali, Hamid Raza, Syed Muzahir Abbas, Leo P. Ligthart

**Affiliations:** 1 School of Information and Electronics, Beijing Institute of Technology, Beijing 100081, China; waseem@bit.edu.cn (W.S.); qasimali@bit.edu.cn (Q.A.); hamidraza@bit.edu.cn (H.R.); 2 Faculty of Science and Engineering, Macquaire University, Sydney 2109, Australia; syed.abbas@mq.edu.au; 3 Faculty of Electrical Engineering, Delft University of Technology, 2628 CD Delft, The Netherlands; l.p.ligthart@gmail.com

**Keywords:** CSRR, metamaterials, sensor

## Abstract

This paper presents a metamaterial sensor using a double slit complementary square ring resonator (DS-CSRR) that has been utilized for the measurement of dielectric materials, especially coal powder. The design is optimized for best performance of deep notch depth in transmission coefficient (Magnitude of S${}_{21}$). Sensitivity analysis of transmission coefficient with respect to structure dimensions has been carried out. Metamaterial properties of double negative permitivity and permeability were extracted from the S–parameters of this sensor. The optimized structure is fabricated using low cost FR-4 PCB board. Measured result shows resonance frequency of 4.75 GHz with a deep notch up to −41 dB. Simulated and measured results show good agreement in desired frequency band. For material characterization, first, two known materials are characterized using this metamaterial sensor. Their respective resonances and dielectric constants are known, so the transcendental equation of the sensor is formulated. Afterwards, the proposed sensor is used for dielectric measurement of two types of coal powder, i.e., Anthracite and Bituminous. The measured value of dielectric constant of Anthracite coal is 3.5 and of Bituminous coal is 2.52. This is a simple and effective nondestructive measurement technique for material testing applications.

## 1. Introduction

Metamaterials (MM) with negative property of permeability was shown experimentally in 1999 as split ring resonators (SRR) [1]. Later, the first complementary split ring resonator (CSRR)-based metamaterial sensor was reported [2]. A split ring resonator is magnetically coupled to transmission lines, such as a Microstrip or coplanar waveguide, while its negative image, complementary split ring resonator, is electrically coupled to these transmission lines.

The CSRR is a metallic negative image of SRR. The equivalent circuit of CSRR is an LC Resonator in which a square ring acts as an inductor while the gaps between rings and their adjacent ground plane act as capacitor. Several parameters affect the resonance frequency and notch depth of proposed sensor such as ring width, gap between rings, and slit size. Energy stored at resonance frequency generates fringing fields. These fringing fields, when perturbed by a dielectric material, can be used to determine the dielectric constant of unknown material.

SRR and CSRR are subwavelength or electrically small metamaterial resonators [3]. The resonance frequency of these resonators is reduced if another material is placed over them. This frequency shift determines the value of the dielectric constant of the perturbed material. CSRR-based sensors are preferred over SRR-based sensors for dielectric measurements because, for the exact same dimensions, CSRR-based sensors exhibit higher resonance frequency shift [4]. This means CSRR has higher dielectric constant sensitivity as compared to SRR. As CSRRs are electrically coupled to a Microstrip line and SRRs are magnetically coupled, CSRRs are more sensitive to permittivity and SRRs are more sensitive to the permeability of perturbed materials. Deep notch in transmission coefficient (S${}_{21}$) aids in improving accuracy of this metamaterial sensor.

Most reported unit cell square and circular CSRRs have same ring width, gap width, and slit width [4,5]. These are single slit sensors having a notch depth of about −16 to −22 dB. Two different widths and gaps in circular and rectangular resonators are reported in [6,7], but their notch depth is also limited to −21 and −27 dB. The proposed sensor exhibits a deep notch by using double slits and optimizing the ring width and gaps. These steps are explained in Section 3. CSRRs are also used to characterize the dielectric constant of liquids [8,9] apart from characterization of materials in solid form.

Efforts have also been focused on employing multiple resonators for deep notch [10,11]. In this work, better performance for transmission coefficient (S${}_{21}$) is achieved by only a single optimized resonator, resulting in better measurement accuracy. A highly sensitive metamaterial sensor is designed which has a deep transmission notch of −41 dB. Low-cost FR-4 material is used for sensor fabrication and a non-destructive evaluation of dielectric constant of coal powder is performed. This technique is non-destructive because physical and electrical properties remained same before and after testing.

At microwave frequencies, several methods can be employed to measure the dielectric constant of coal including resonant cavity [12], coaxial and waveguide method [13], and free space method [14]. The dielectric constant of coal is a function of its coal rank [12], moisture content [13], temperature [15], and frequency of operation [13]. This paper demonstrates the utilization of double slit complementary split ring resonator (DS-CSRR)-based MM sensors for characterization of the dielectric constant of two ranks of coal. DS-CSRR resonance frequency has twice the value as compared to resonance frequency of single slit CSRR [16]. A summary of dielectric measurement methods of different materials is listed in Table 1. Two types of coal powder are used in this work, Anthracite and Bituminous. Anthracite has carbon content of more than 90% while Bituminous has 60–80% carbon content. The proposed sensor is used in C-band, while X-Band [13] and terahertz frequency characterization of coal is presented in [14].

In this paper, initially, an optimized structure of CSRR is designed in C-Band and characterized with known dielectric materials to formulate the sensor’s characteristic equation. Afterwards, this CSRR sensor is used to characterize the dielectric constant of coal powder. The measured results show the uniformity with the previous work as the dielectric constant of coal increases with coal rank [12]. Therefore, the focus of this paper is to design a simplified DS-CSRR and show its utilization as a new method for measurement of dielectric constants of coal powder.

## 2. Design and Simulations

The proposed sensor is very compact in size and it is designed on easily available commercial material FR-4. The dielectric constant of used FR-4 is 4.4, the PCB height is 0.8 mm, and 17 μm copper is used on both sides. The top sides consist of a Microstrip line, while CSRR is etched by chemical etching on the ground plane. The dimensions of the proposed sensor are (24 × 60) mm.

**Figure 1:**
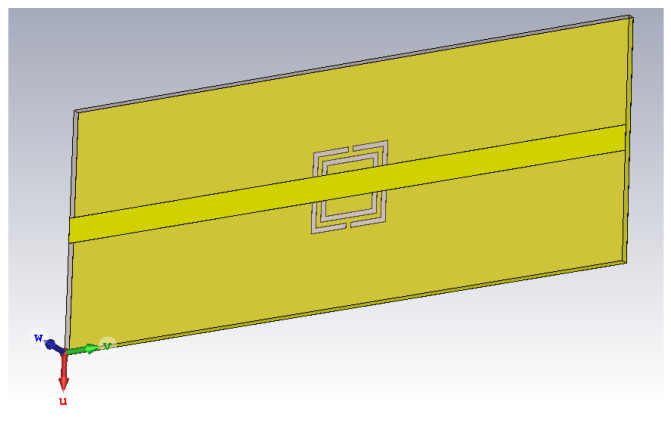
Proposed sensor’s perspective view with PCB substrate hidden.

**Figure 2:**
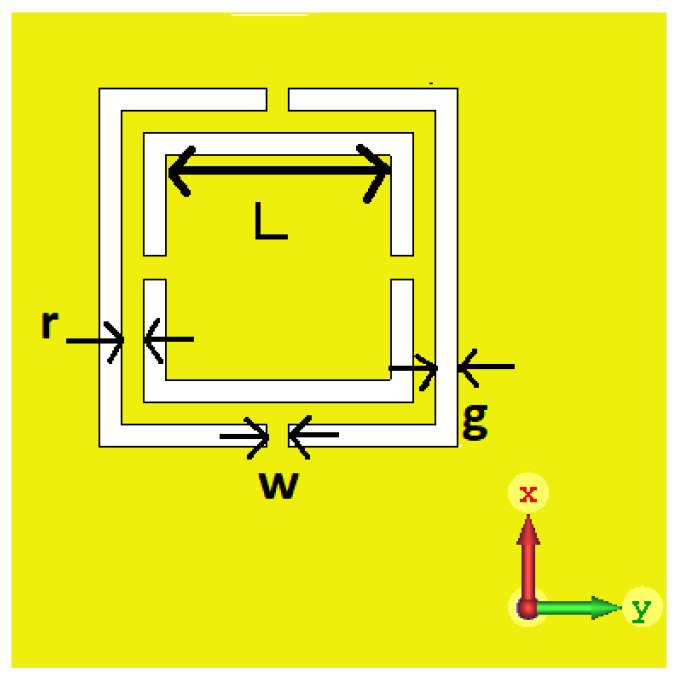
Structure of complementary square ring resonator (resonant structure) (figure modified).

The notch present in transmission parameter (S${}_{21}$) of this two port sensor is known as its resonant frequency. The frequency value of this notch determines the value of dielectric constant of material and its magnitude value determines the accuracy of the sensor. Resonance frequency Equation (1) and Q-factor (2) of a CSRR can be found from its equivalent circuit [8]. The structure of proposed sensor, as shown in Figure 1, is designed to give resonance frequency in C-band at 4.85 GHz. Microstrip Line has width of 2.5 mm while square resonator has length (L) 5 mm, ring-width (r) 0.5 mm, slit-width (w) 0.5 mm, and gap (g) of 0.5 mm. A close-up view of the CSRR structure indicating structure parameters is shown in Figure 2.

For obtaining a deep S${}_{21}$ notch, i.e., −40 dB, gaps between rings become unrealizable or impractical to fabricate using this low cost material FR-4 if resonator length is designed using single slits, so DS-CSRR is used. The deep resonance value in transmission coefficient S${}_{21}$ in the resonance band is desired because it ensures the accurate resonance frequency measurement. This value is then utilized in a transcendental equation to find the dielectric constant of Material Under Test (MUT).

(1) $$f_{r}=\frac{1}{2\pi \sqrt{L_{r}\left(C_{s}+C_{r}\right)}}$$

This resonance has Q-factor equal to

(2) $$Q=R\sqrt{\frac{\left(C_{s}+C_{r}\right)}{L_{r}}}$$

**Figure 3:**
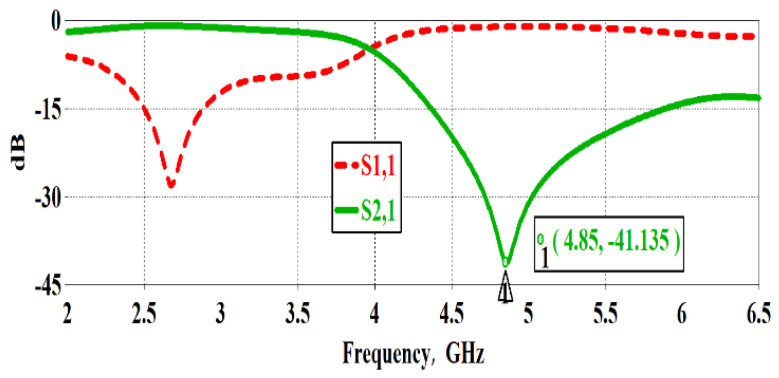
Simulated S-parameters of proposed DS-CSRR.

Simulations are carried out using CST microwave studio to see the S-parameters’ response. A stop band behaviour with deep notch is observed at the resonance frequency of 4.85 GHz. A sharp notch of −41 dB is achieved after multiple simulations. The simulated S-parameter results of DS-CSRR-based sensor are shown in Figure 3.

## 3. Parametric Analysis

**Figure 4:**
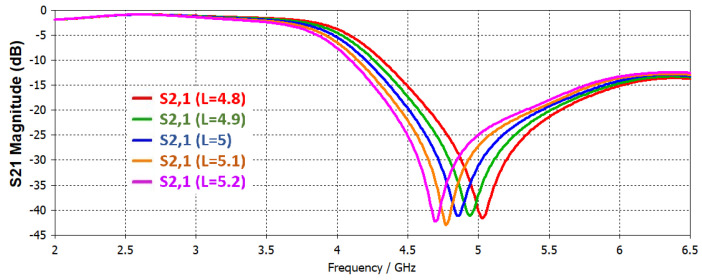
Simulated S${}_{21}$ in case of variation in resonator length (L).

**Figure 5:**
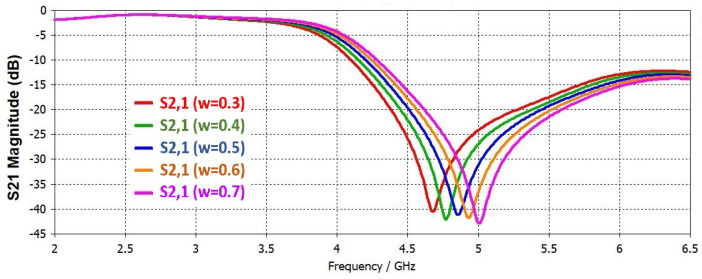
Simulated S${}_{21}$ in case of variation in slit width (w).

As widely reported [5,6,7,8,9], if the length (L) of resonator is increased the resonance frequency will be decreased. Length is changed from 4.7 to 5.2 mm, and simulation results are shown in Figure 4. If the gap (g) is decreased between the ring and adjacent ground plane, the resonance frequency will increase. By increasing the width of central ring the resonant frequency increases in the same way as the slit width (w). Slit width is changed from 0.3 to 0.7 mm, and corresponding S${}_{21}$ results are shown in Figure 5. A single parameter is changed at a time while other parameters are kept constant.

**Figure 6:**
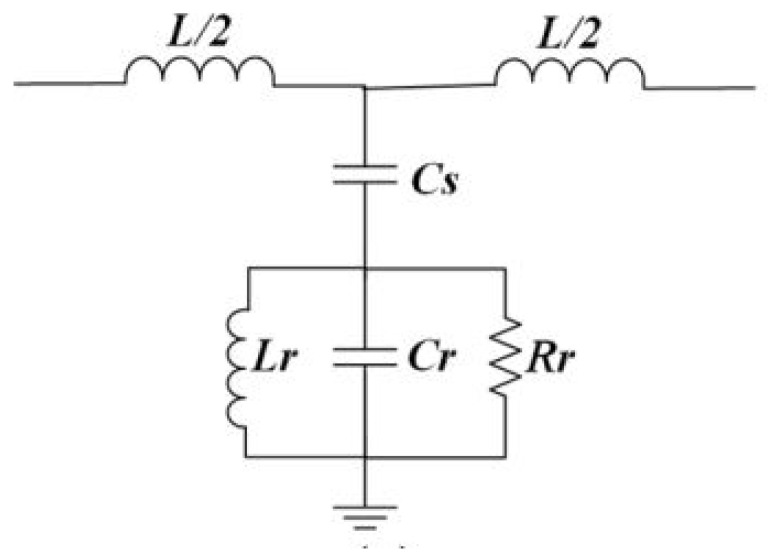
Equivalent circuit of CSRR.

During the design, two options of double slits are considered. One is outer horizontal slits aligned with the top Microstrip line, while the other is vertical outer slits perpendicular to the top Microstrip line. It is noted that the outer vertical slits show a deeper notch in S${}_{21}$ as compared to the outer horizontal slits. This analysis is summarized in Table 2. The equivalent circuit model of CSRR sensor is of a shunt LCR resonator coupled to the Microstrip line by a capacitance Cs [5] shown in Figure 6. This shunt resonator is a parallel combination of Lr, Cr, and Rr. Using the procedure given in [5], CSRR equivalent parameters Cr, Lr, and Rr are calculated to be 0.82 pF, 0.46 nH, and 1450 Ohm, respectively. Calculated coupling capacitance Cs is 1.52 pF and L is 2.3 nH.

**Figure 7:**
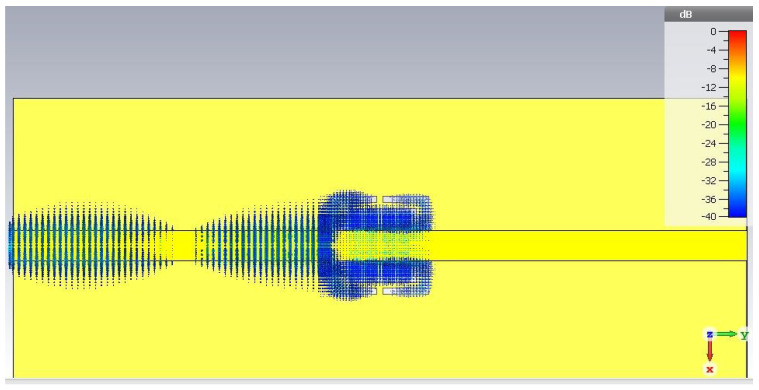
E-field at resonance frequency.

Simulations of this sensor are also performed by placing three standard dielectric materials on top of resonant structure, namely Rogers^®^ corporation RO5870 and RO4003C and commercially available FR-4. Sample size of MUT in simulation is (7 × 7 × 2) mm${}^{3}$, chosen to completely surround the resonant structure. After the optimization steps, E-field at resonance is studied as shown in Figure 7. Tangential E-field excited the CSRR structure. Almost all the signal is absorbed by the resonant structure as the transmission notch depth is −41 dB.

**Figure 8:**
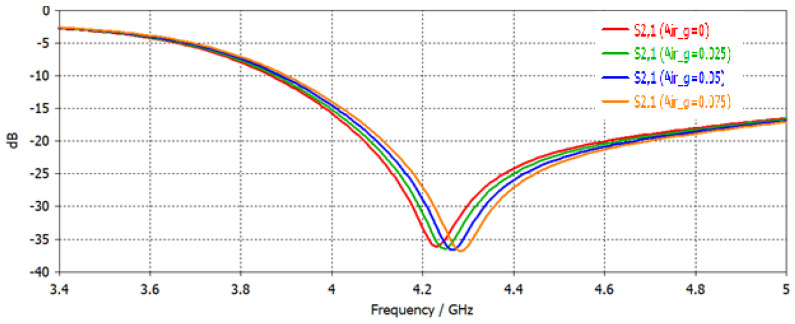
Analysis of air gap effect on the resonance frequency.

Two factors which can add inaccuracy in the resonance frequency measurement are the air gaps between the sensor and known MUTs and secondly dielectric loss [18] or loss tangent. These factors are simulated and their effect is studied. Air gaps are like adding a material between the sensor and MUT. As the air has the lowest dielectric constant it has higher resonance frequency. Air gaps of 0 to 75 μm are added between the sensor and MUT. The results are shown in Figure 8. As evident from this simulation, as the air gap is increased the resonance frequency is increased or shifted to the right.

**Figure 9:**
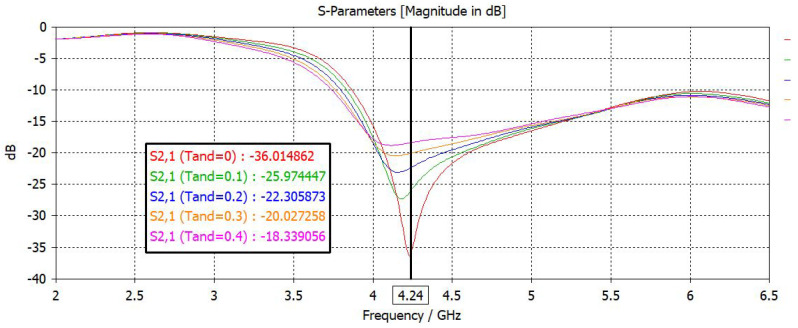
Analysis of loss tangent (Tand) variation on the resonance frequency.

Loss tangent also affects the resonance frequency, but in the case of MUTs used in simulation and measurement little effect is noted. A simulation is carried out to investigate the effect of loss tangent on resonance frequency. Loss tangent is varied from 0 to 0.4 with step size of 0.1, and resulting graphs are shown in Figure 9. As evident from results, dielectric loss can decrease the notch depth at resonance frequency, and it also shifts the resonance frequency. This will add inaccuracy to resonance frequency measurements, but low loss materials used in the measurement have little effect on resonance frequency, and their dielectric loss contribution is minimal. Higher dielectric loss means reduced sensitivity. As all known MUTs used in the measurement have a loss tangent less than 0.1, little change is expected in resonance frequency.

## 4. Fabrication and Characterization

**Figure 10:**
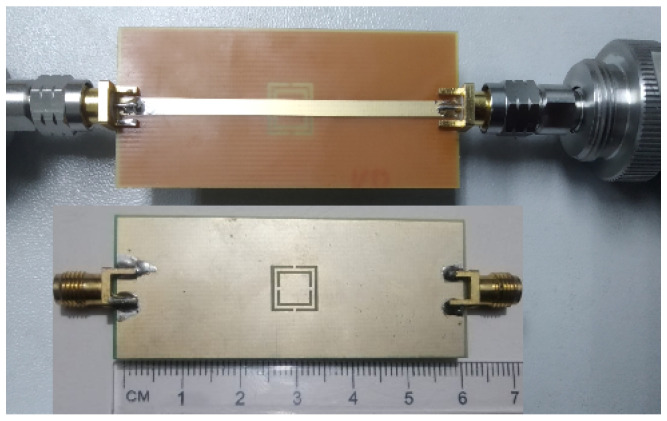
Fabricated prototype of the proposed sensor.

**Figure 11:**
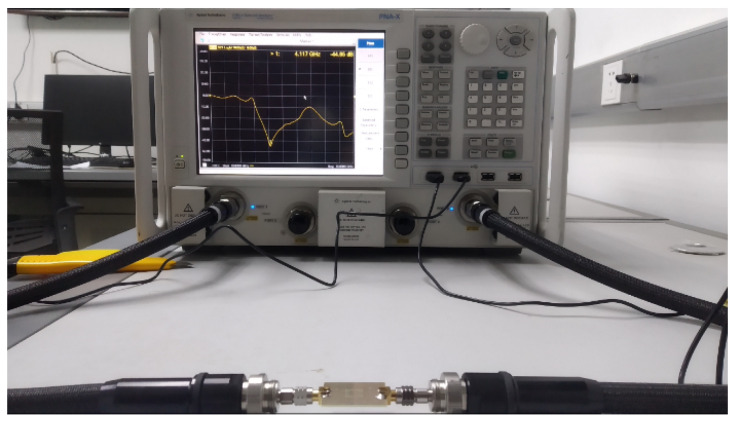
Measurement of proposed DS-CSRR-based sensor using network analyser N5224B.

The proposed sensor is fabricated using standard low cost FR-4 material having 17 μm copper on both sides of the PCB. Standard SMA connectors are used to interface with the network analyser as shown in Figure 10. Resonance frequency of this sensor is measured, first without any material, using Keysight technologies network analyser PNAX–N5224B as depicted in Figure 11.

**Figure 12:**
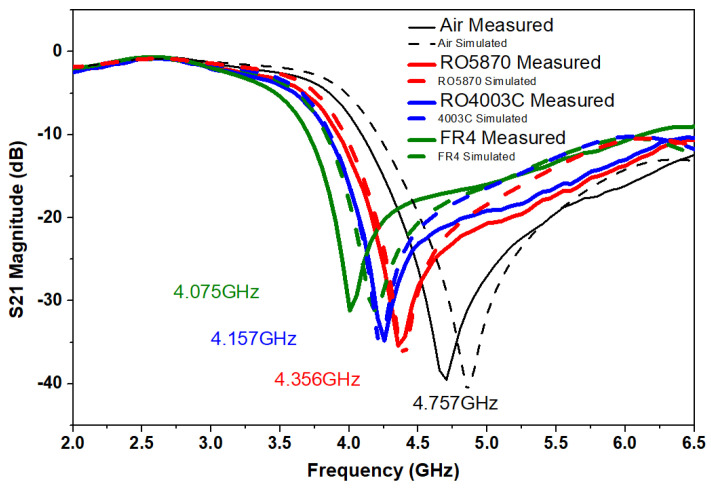
S${}_{21}$ Measured results of metamaterial sensor with standard materials. Solid lines indicate measured results and dashed lines indicate simulated results.

This resonance frequency is called air dielectric resonance frequency. This sensor exhibits a deep notch of −40.5 dB as anticipated in simulated results. Table 3 summarizes the comparison between similar published structures to compare its performance, as all these sensors are used in conjunction with the Microstrip line. As evident from this comparison, the DS-CSRR sensor can be used as a preferred method for dielectric measurements. During measurement, the proposed sensor is then perturbed using three standard dielectric materials, RO5870, RO4003C, and FR-4. These materials are placed on top of the DS-CSRR resonant structure. Their respective resonant frequencies are recorded and shown in Figure 12.

Air dielectric has the highest resonance frequency while the resonance frequency keeps shifting to the lower side as the dielectric constant of perturbed MUTs increases. A comparison of respective simulated and measured resonance frequencies of MUTs is shown in Table 4. It is evident from Figure 12 that RO5870 has lowest loss tangent and FR-4 has highest loss tangent among the tested materials-based on their respective notch depth. Notch depth decreases as the loss tangent of MUT increases.

The resonant frequencies of these standard materials are used to derive the characteristic equation of this sensor known as the transcendental equation [11]. The resonant frequency of this sensor can be approximated by second order polynomial equation. This is a parabolic approximation for the resonant frequency equation of the test material represented in (3).

(3) $$f_{r,MUT}=A_{1}+A_{2}\left(\epsilon_{r}^{{}^{\prime }}-1\right)+A_{3}{\left(\epsilon_{r}^{{}^{\prime }}-1\right)}^{2}$$

(4) $$f_{r,MUT}=4.75-0.3659\left(\epsilon_{r}^{{}^{\prime }}-1\right)+0.049{\left(\epsilon_{r}^{{}^{\prime }}-1\right)}^{2}$$

(5) $$\epsilon_{r}^{{}^{\prime }}=\frac{0.3659-\sqrt{0.1338-0.196(4.75-f_{r,MUT})}}{0.098}+1$$

During the solution of Equation (3), *A*${}_{1}$ is the resonant frequency when $\epsilon_{r}^{{}^{\prime }}$ is taken as 1 (the case of air dielectric). *A*${}_{2}$ and *A*${}_{3}$ are found as a result of two equations, when $\epsilon_{r}^{{}^{\prime }}$ is replaced by two standard MUTs dielectric constants in Equation (3). Therefore, Equation (4) is formed by the solution of Equation (3) using measured resonance frequencies of two standard materials. Resonance frequencies of standard materials are recorded during measurement as shown in Figure 12. The solution of the parabolic equation for finding the dielectric constant of unknown MUT is represented by Equation (5). This equation can also be used to measure the dielectric constant of unknown material when perturbed by using this metamaterial sensor.

**Figure 13:**
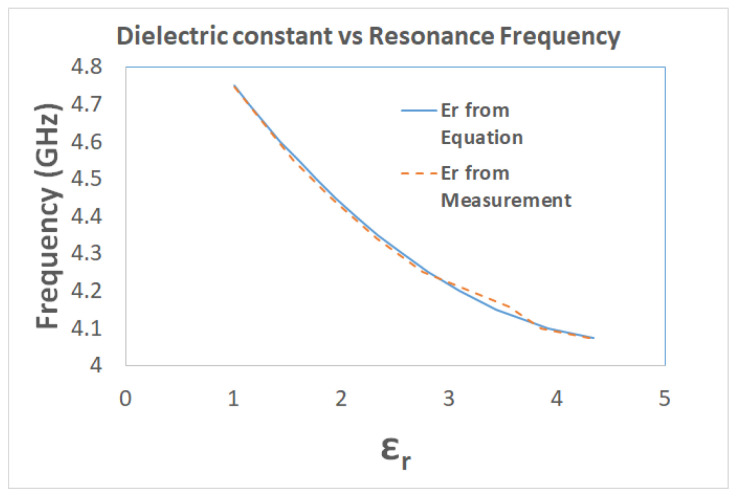
Comparison of resonance frequency vs Er calculated from equation and of known MUTs.

The accuracy of resonant frequency measurement is directly related to the deep notch exhibited by the DS-CSRR sensor. If the notch in transmission coefficient is not deep, it can result in reading a less accurate resonant frequency of the sensor itself and the sample materials. This will lead to a characteristic equation whose accuracy will not be up to the mark and a less accurate calculation of the unknown material’s dielectric constant. The characteristic equation of the sensor gives the calculated dielectric constant of unknown MUT for any given value of resonance frequency. Results from (5), compared with the known values of dielectric constant of MUTs, are shown in Figure 13.

## 5. Characterization of Coal

**Figure 14:**
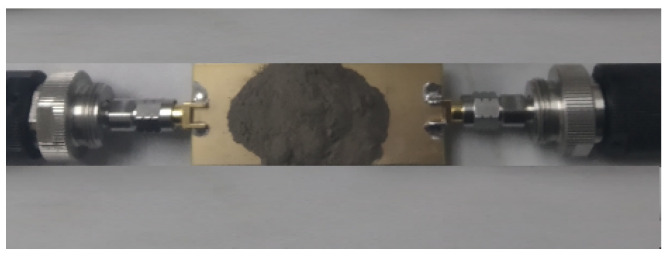
Anthracite sample testing with network analyser.

**Figure 15:**
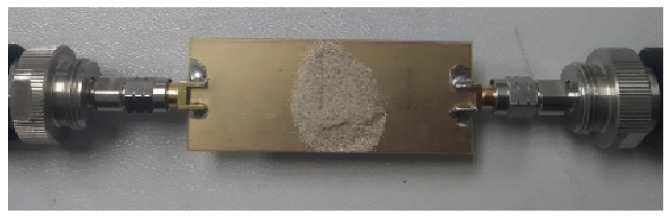
Bituminous sample testing with network analyser.

**Figure 16:**
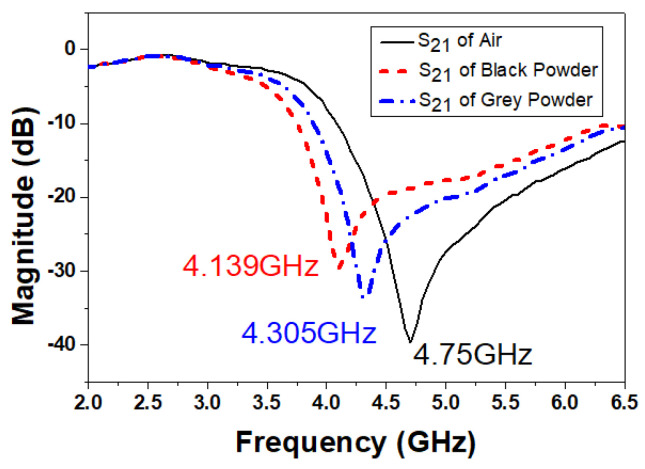
Measured $S_{21}$ of Anthracite (black powder) and Bituminous (grey powder).

Two China-based coals, namely Guizhou Anthracite and Jilin Bituminous, are tested subsequently as shown in Figure 14 and Figure 15, respectively. As the carbon content percentage or coal rank is increased the dielectric constant is increased [12]. Coal powder is poured on top of the resonant DS-CSRR structure, completely surrounding it. Coal powder is poured on the DS-CSRR sensing area, exceeding each side by 2 mm. During measurement, it is noted that if we pour more powder, exceeding the sensor area by 3 mm, 4 mm, or more, the results are same as previous readings (2 mm). Fringing fields which are confined nearby the sensing area are used to measure the dielectric constant. The respective transmission coefficients indicating resonance frequencies are recorded and shown in Figure 16.

Anthracite has a higher coal rank (carbon content > 90%) than Bituminous coal (carbon content 60–80%). Guizhou Anthracite has resonant frequency of 4.139 GHz and Jilin Bituminous has 4.305 GHz. In the case of Anthracite, the resonant frequency is lower (or farthest away from the air resonance of sensor) than that of the Bituminous, implying that former coal powder has a higher value of dielectric constant.

Using (5), the respective dielectric constants of coal powder are calculated. The dielectric constant of Anthracite powder is found to be 3.5, while Bituminous has a dielectric constant of 2.52. The trend of these values is the same as reported in the literature [12,19]; as the coal rank increases, the dielectric constant increases. As temperature and humidity affects the dielectric constant of coal powder [14], these measurements are taken in a controlled indoor environment of 20 °C temperature and relative humidity of 65%.

This dielectric characterization method employing a low cost planar sensor is very simple and cost effective as compared to other bulky and expensive methods such as resonant cavity [12], coaxial and waveguide [13], and free space methods [14]. The dielectric constants of Anthracite and Bituminous coal found by this method are consistent with the carbon content of these respective coals. It is also demonstrated that utilization of double slit complementary split ring resonator (DS-CSRR)-based MM sensor for characterization of dielectric constant can yield accurate results due to the deep notch of S${}_{21}$.

## 6. Conclusions

A double slit CSRR metamaterial sensor has been presented as a preferred method for coal’s dielectric constant measurement. The proposed design is a simple and low cost solution. The design of this sensor, with detailed parametric analysis, is given. A deep transmission notch depth of −41 dB is achieved, which has the advantage of higher sensitivity and better resolution in resonance frequency and magnitude. After fabrication, three standard materials are placed on this sensor and their respective resonance frequencies are recorded. Then, these resonance frequencies and their respective dielectric constants are used to formulate the characteristic equation of this sensor. Afterwards, the sensor is used to measure the dielectric constant of Anthracite and Bituminous coal. Conversely, this CSRR-based sensor can be used to distinguish between the Anthracite and Bituminous coal. Apart from coal powder, testing it can be used in applications including dielectric properties measurement and non-destructive testing of materials.

## Figures and Tables

**Table 1:** Dielectric Properties Measurement techniques.

Ref.	Resonator	Frequency	Measurement	Sensor	Material
	Type	Band	Process	Size	Type
[4]	Square CSRR	L	Simple	Small	Solids
[5]	Circular CSRR	L,S,C	Simple	Moderate	Solids
[6]	Circular CSRR	S	Simple	Small	Solids
[7]	Rectangular CSRR	S	Simple	Small	Solids
[8]	Square CSRR	S	Simple	Small	Liquid
[9]	Square CSRR	S	Simple	Small	Liquid
[10]	Planar Horn and rings	S	Simple	Small	Fungi
[11]	Spiral CCSR	S	Simple	Small	Solids
[17]	Square CSRR	S	Simple	Small	Liquid
[12]	Resonant cavity	L,S	Complex	Large	Coal
[13]	Waveguide	X	Complex	Large	Coal
[14]	Free Space	Terahertz	Complex	Large	Coal
Proposed	Square DS-CSRR	C	Simple	Small	Coal

**Table 2:** Parametric analysis of the proposed sensor.

Parameter	Description	Optimized Value	Change	Resonance Frequency
r	Ring width	0.5 mm	Increase	Increased
g	Gap width	0.5 mm	Decrease	Increased
w	Slit width	0.5 mm	Increase	Increased
L	Resonator width	5 mm	Decrease	Increased

**Table 3:** Performance comparison of metamaterial Sensors.

Reference	Resonator Type	Number of Resonators	S${}_{21}$ Notch Depth
[4]	Single Slit Square CSRR	1	−16
[17]	Single Slit Square CSRR	1	−24
[5]	Single Slit Circular CSRR	1	−22
[7]	Single Slit Rectangular CSRR	1	−27
[8]	Single Slit Square CSRR	1	−25
[9]	Single Slit Square CSRR	1	−28
[10]	Complementary Horn and U-shaped rings	2	−24
[11]	Spiral CCSR	2	−30
This Work	DS-CSRR	1	−40.5

**Table 4:** Comparison of simulated and measured results with three standard materials under test (MUT).

Material Under Test	Frequency (GHz)			Difference %
	Simulated	Measured	Difference	
Air	4.85	4.75	0.1	2.06
RO5870	4.4	4.35	0.05	1.13
RO4003	4.24	4.157	0.09	2.12
FR-4	4.21	4.075	0.13	3.08
